# Explainable artificial-intelligence-based hyperspectral image analysis for leaf disease detection in intercropping system

**DOI:** 10.3389/fpls.2026.1789542

**Published:** 2026-04-01

**Authors:** Varun Malik, Asma AlJarullah, Tahani Alsubait, Amna Ikram, S. B. Goyal, Mudassir Khan

**Affiliations:** 1 Chitkara University Institute of Engineering and Technology, Chitkara University, Rajpura, Punjab, India; 2 Department of Informatics and Computer Systems, King Khalid University, Abha, Saudi Arabia; 3 Department of Computer Science and Artificial Intelligence, College of Computing, Umm Al-Qura University, Makkah, Saudi Arabia; 4 Department of Computer Science and IT, Government Sadiq College Women University, Bahawalpur, Pakistan; 5 Department of Computer Science, College of Computer Science, Applied College Tanumah, King Khalid University, Abha, Saudi Arabia; 6 Jadara University Research Center, Jadara University, Irbid, Jordan

**Keywords:** disease pattern analysis, feature selection, maize-soybean, pea-cucumber, precision agriculture, spectral-spatial features

## Abstract

**Introduction:**

Intercropping regimes enhance the efficiency of land use and ecological sustainability but present serious problems to automated disease analysis since the overlapping canopy and the similarity of symptoms in crop species are visually indistinguishable.

**Methods:**

This work presents an explainable artificial intelligence (XAI)-based hyperspectral analysis on leaf disease in intercropping systems. The framework combines the spectral–spatial feature generators that utilize transformers including vision transformer (ViT), Swin transformer, pyramid vision transformer (PVT), and detection transformer (DETR) to identify nuanced biochemical and structural changes in crop combinations for maize–soybean and pea–cucumber. In order to reduce spectral redundancy and high dimensionality, an enhanced greedy political optimization (EGPO) algorithm is used as a wrapper-based feature selection strategy. A capsule spatial shift neural network (CSSNet) is used to predict the classification of diseases. Explainable AI methods, such as Local Interpretable Model-agnostic Explanations (LIME) and SHapley Additive exPlanations (SHAP) feature attribution analysis and gradient-weighted class activation mapping (Grad-CAM) visualization of disease-relevant regions, provide model transparency. The DETR + EGPO + CSSNet framework is tested on the conventional feature selection methods.

**Results and discussion:**

The results or findings on publicly available hyperspectral datasets on intercropping show an average recall of 99.998% with high region consistency (Dice score: 99.997%) of activation maps and expert-marked disease regions. These findings affirm that the proposed framework is highly accurate, stable, and interpretable to identify subtle and overlapping disease in leaves in a complex system of intercropping.

## 1 Introduction

Crop production directly affects human nutrition and economic stability, and agriculture is the foundation of both environmental sustainability and global food security. Due to their potential to increase land productivity, improve soil fertility, and preserve ecological balance, intercropping systems—in which several crops are grown concurrently on the same piece of land—are becoming more and more popular (Mahlayeye et al., 2024; Wang Z. et al., 2025). However, because of overlapping canopies, intricate microclimates, and the presence of several diseases with comparable visual signs, these systems provide particular difficulties for crop health monitoring (Wang W. et al., 2025). Leaf disease is one of the most dangerous biotic stresses which can reduce crop yields by as much as 40% (Xiao et al., 2026). Sustainable agriculture and food security depend on the early detection and management of these diseases, particularly in developing nations where smallholder farmers are disproportionately impacted (Yang L. et al., 2026). Conventional disease detection techniques rely on manual sampling and visual inspection, which are labor-intensive, time-consuming, and subject to subjective errors (Li et al., 2026; Veena et al., 2024). By capturing high-resolution spectral information over several wavelengths for every spatial pixel, hyperspectral imaging makes it possible to identify minute biochemical and structural alterations in plant leaves (Rahman et al., 2025b; Viaud et al., 2025). Hyperspectral data offer deeper information than red, green, and blue (RGB) images, which are utilized with algorithms for accurate disease identification (Fan et al., 2026). Recent research has identified plant illnesses by analyzing hyperspectral pictures using machine learning (ML) and deep learning (DL) models. For feature classification in monoculture and polyculture systems, support vector machines (SVM), random forest (RF), k-nearest neighbors (k-NN), and artificial neural network (ANN) have been used (Moreira et al., 2026; Wang YM. et al., 2026). Convolutional neural networks (CNNs) used to automatically learn hierarchical features from hyperspectral and multispectral images, achieving higher classification accuracy than conventional, manually developed features such as SIFT, SURF, and HoG (Iqbal et al., 2025; Khaliq et al., 2025). Graph-based models have been investigated to capture spatial–spectral correlations in heterogeneous intercropping environments (Wang L. et al., 2026; Yang H. et al., 2026). Explainable AI techniques (XAI), such as locally interpretable model-agnostic explanations (LIME), Shapley additive explanations (SHAP), and gradient-weighted class activation maps (Grad-CAM), have been used to highlight critical spectral and spatial regions that influence classification decisions for diseases (Liu Z. et al., 2026; Oladele et al., 2026). These methods enable stakeholders to understand the logic behind the model’s predictions, thereby fostering trust and facilitating actionable decisions in precision agriculture (Liu Y. et al., 2026).

The reliable detection of leaf diseases in complex intercropping systems remains challenging due to high spectral dimensionality, overlapping vegetation, and varying climatic conditions. This study aims to build on existing research by using hyperspectral images, ML/DL, and XAI techniques to enhance the early detection of leaf diseases in intercropping systems, supporting sustainable crop management and precision agriculture. XAI-based hyperspectral image analysis is proposed for leaf disease detection in intercropping systems. The major contributions of the proposed work are given as follows:

1. The vision transformer models—including vision transformer (ViT), Swin transformer (STrans), pyramid vision transformer (PVT), and detection transformer (DETR)—are used to extract spectral–spatial features from hyperspectral leaf images.
2. To improve computational efficiency, the enhanced greedy political optimization (EGPO) algorithm is used for feature selection and dimensionality reduction.
3. The capsule spatial shift neural network (CSSNet) is used to classify leaf diseases while preserving spatial hierarchies and relationships in hyperspectral images.
4. XAI techniques—LIME and SHAP—are incorporated to provide insight into the model’s decision-making process, detecting which spectral–spatial features most influence disease predictions. Grad-CAM is used to generate visual explanations and highlights critical regions in leaf images that contribute to classification, thereby improving trust, transparency, and interpretability of the system for end-users such as agronomists and farmers.
5. The proposed framework is tested on hyperspectral datasets from maize–soybean and pea–cucumber intercropping in multi-crop agricultural environments.

The paper is organized as follows: Section 2 reviews related work on hyperspectral leaf disease detection. Section 3 describes the materials and methods, including feature extraction, feature optimization, classification, and interpretability. Section 4 presents results and discussion on maize–soybean and pea–cucumber datasets. Section 5 concludes the study, summarizing key findings and implications for real-time, interpretable disease detection in intercropping systems.

## 2 Related work

Soybean/maize intercropping was used to increase the rhizosphere microbial communities, which increases the utilization of carbon and the expression of functional genes such as acsH and nosZ1 (Gao et al., 2024). There was enhanced soil nutrient value, such as organic carbon, nitrogen, phosphorus, and sulfur, by cotton–legume intercropping (Dhanorkar et al., 2025). The intercropping of tomatoes/potatoes–onion had an effect on root distribution and microbial diversity, improving nutrient availability and yield and decreasing the diseased fruits (He X. et al., 2025). Intercropped indica–japonica rice exhibited superior growth of leaf area and yield compared to monocropped rice, owing to the complementary growth trends in 2 years (Nasar et al., 2025). In the intercropping of peanut/maize, it has been reported that root exudates have an effect on the cycling of nitrogen and gene expression (Si et al., 2025). Intercropping of wheat and chickpea enhanced the biological fixation of nitrogen and phosphorus (Rahman et al., 2025a). Peanut/cotton intercropping increased the keystone microbial groups that promote soil fertility (Zhang et al., 2026). Intercropping wheat/alfalfa enhanced the water productivity and yield and decreased the water utilization (Ma L. et al., 2025). Cotton intercropping ensured the stable quality of the fibers in the face of environmental stress (Qin et al., 2025). In pineapple intercropping with YOLO-NAS-L, it was found to be characterized by high fruit recognition accuracy but failed to evaluate leaf health (Hong et al., 2025). Hyperspectral imaging of maize and intercropping soybean, with the DBO-BiLSTM model and the CARS spectral feature selection, yielded an accuracy level of 98.7% (Liu et al., 2024), and hybrid DL models with deep convolutional networks and phase attention networks (PANet) demonstrated a greater accuracy level of over 99% on peas, cucumbers, maize, and soybean (Goyal et al., 2025), which demonstrates the possibility of hyperspectral imaging and deep learning in automated disease detection. Although these results are promising, there are a number of limitations in practical intercropping systems (Syed et al., 2025b; Syed et al., 2019; Syed et al., 2025a): the majority of the models are single-crop intensive or even to fruit levels of phenotyping, leading to decreased applicability to multi-species intercropping systems; early disease detection and real-time monitoring are not often developed; and model predictions are difficult to interpret, lowering their practical application. Moreover, hyperspectral problems exist in practice in intercropping, including spectral mixing, shadowing, occlusion, and complex canopy forms, which have not been reviewed or sufficiently tackled. In order to address these shortcomings, emerging studies focus on high-resolution, non-destructive methods that integrate hyperspectral imaging with CNNs, transformers, and explainable AI (XAI), which allow the effective early detection of leaf diseases, visualization of diseased areas, and consistent implementation across multi-species intercropping systems. In spite of the fact that intercropping enhances agronomics performance, the existing leaf disease monitoring solutions (**Table 1**) are not sufficient, such as destructive sampling, visual gradient, or post-harvest analysis, and non-destructive, real-time, explainable ML/DL models are eagerly needed to detect early-stage leaf diseases in complex intercropping systems (Gao et al., 2024; Liu et al., 2024; Dhanorkar et al., 2025; Goyal et al., 2025; He X. et al., 2025; Hong et al., 2025; Ma L. et al., 2025; Nasar et al., 2025; Qin et al., 2025; Rahman et al., 2025a; Si et al., 2025; Zhang et al., 2026).

**Table 1:** Summary of intercropping studies related to leaf disease detection models in intercropping systems.

Ref.	Intercrops	Models	Dataset	Key findings	Observed gap	Relevance to current work
(Gao et al., 2024)	Soybean/maize	DNA-SIP, high-throughput sequencing	Pot experiment, rhizosphere soil	Functional gene expression ↑ (acsH, nosZ1)	Focused on below-ground microbial interactions	CNN/transformer with hyperspectral data for early leaf disease detection
(Dhanorkar et al., 2025)	Cotton + legume	Soil chemical analysis	Field soil samples	SOC ↑27%, N ↑35%, P ↑45%, S ↑39%	No leaf-level disease monitoring	Vision-based hyperspectral detection at leaf level
(He X. et al., 2025)	Tomato/potato–onion	qPCR, Illumina MiSeq, RDA	Rhizosphere soil	Improved root distribution and yield; diseased fruits ↓	Limited to below-ground microbial analysis	DL with spectral–spatial features for early disease localization
(Nasar et al., 2025)	Indica–japonica rice	Field experiment	Field growth data	Leaf area ↑ (indica 45%, japonica 46%), yield ↑ (indica 18.5%)	No automated leaf disease detection	Hyperspectral + CNN/transformer for leaf disease detection
(Si et al., 2025)	Peanut/maize	Metabolomics, transcriptomics	Rhizosphere soil	Root exudates influence N-cycle; DEGs ↑1,036/↓797	No spectral or image-based disease detection	CSSNet + hyperspectral features for leaf disease identification
(Rahman et al., 2025a)	Wheat/chickpea	^15^N labeling, field experiment	Pot experiment	BNF ↑11%–323%, P uptake ↑	Non-destructive leaf disease detection	Hyperspectral imaging + XAI for real-time detection
(Zhang et al., 2026)	Peanut/cotton	Co-occurrence network, BNF	Field soil samples	Keystone microbial taxa enriched 78%–85%	Above-ground leaf health not assessed	Transformer + CNN for leaf disease localization
(Ma L. et al., 2025)	Wheat/alfalfa	Field experiment	Field experiment	Yield ↑8.7%–31%, WU ↓1.1%–13.1%, WP ↑0.1%–24%	No image-based disease monitoring	Hyperspectral imaging for early disease detection
(Qin et al., 2025)	Cotton intercropping	5-year field study	Long-term fiber data	Fiber length stable; yield affected by stress 17%–33%	Leaf disease monitoring missing	CNN/transformer + XAI for leaf disease detection
(Hong et al., 2025)	Pineapple intercropping	YOLO-NAS-L	Field images (RGB + 3D)	Fruit recognition accuracy 95.4%	Focused on fruit phenotyping; no leaf disease detection	Hyperspectral + CSSNet for leaf-level disease detection

↑ increased and ↓ reduced.

## 3 Materials and methods

**Figure 1:**
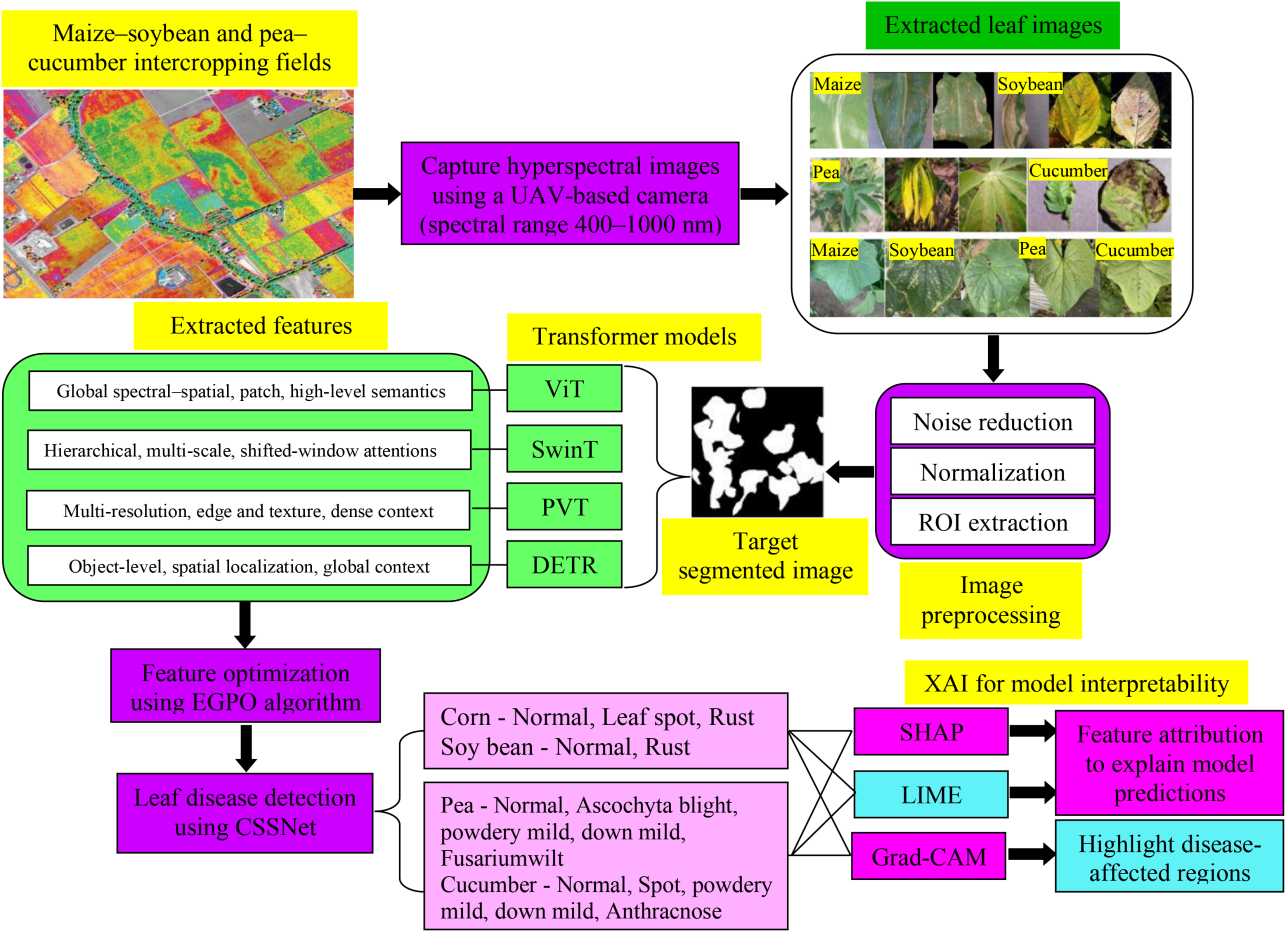
XAI-based hyperspectral image analysis for leaf disease detection in the intercropping system.

**Figure 1** shows the conceptual structure of XAI-based hyperspectral image processing that is suggested to diagnose leaf diseases in intercropping systems. The publicly available hyperspectral datasets were used to conduct the experimental evaluation based on intercropping systems of maize–soybean and pea–cucumber (Liu et al., 2024; Goyal et al., 2025) as compiled by Liu et al. The hyperspectral images of maize and soybean leaves were collected on September 13, 2023 from intercropping fields in Fei Cheng City, Tai’an City, Shandong Province, China. It has 11,006 hyperspectral images obtained with UAV-mounted hyperspectral sensors that have a spectral band of 400–1,000 nm. Each sample was annotated by expert plant pathologists according to the morphology of the lesions, color distribution, spatial distribution, and the nature of the infection, and these annotations were validated ground-truth class labeling. The dataset consists of 1,949 maize images (700 healthy, 630 leaf spot, 644 rust, and 560 combined infections) and 1,260 soybean images (672 healthy, 588 rust) included in the maize–soybean subset. The pea–cucumber subset will have 3,138 pea pictures (700 healthy, 630 *Ascochyta* blight, 616 powdery mildew, 602 downy mildew, and 588 anthracnose). **Table 2** provides a summary of the class distribution and partitioning of databases. Hyperspectral imaging is a process that records changes in physiology manifested in the visible (450,700 nm) and the near-infrared spectroband (700,740 nm), which are essential in making distinctions between diseased and healthy tissues. Healthy leaves normally have high chlorophyll uptake at the range of 450–500 nm and 680–700 nm. By comparison, disease conditions cause quantifiable shifts in reflectance:

**Table 2:** Statistical description of the hyperspectral dataset for maize–soybean and pea–cucumber intercropping fields in Fei Cheng City, Tai’an, Shandong Province, China.

Crop	Disease	Number of samples				Key wavelengths (nm)
		Training	Validation	Testing	Total	
Maize	Healthy	500	100	100	700	450–500, 680–700
	Leaf spot	450	90	90	630	540–580, 700–740
	Rust	460	92	92	644	550–600, 720–740
	Combined infection	400	80	80	560	540–580, 680–740
Soybean	Healthy	480	96	96	672	450–500, 680–700
	Rust	420	84	84	588	550–600, 710–730
Pea	Healthy	500	100	100	700	450–500, 680–700
	*Ascochyta* blight	450	90	90	630	530–570, 700–730
	Powdery mildew	440	88	88	616	550–600, 710–740
	Downy mildew	430	86	86	602	540–590, 700–730
	*Fusarium* wilt	420	84	84	588	550–600, 710–740
Cucumber	Healthy	500	100	100	700	450–500, 680–700
	Angular leaf spot	450	90	90	630	540–580, 710–740
	Powdery mildew	440	88	88	616	550–600, 710–740
	Downy mildew	430	86	86	602	540–590, 700–730
	Anthracnose	420	84	84	588	550–600, 720–740
Total	—	7,110	1,422	1,422	11,006	—

- Maize leaf spot and rust: 540,580 nm and 720,740 nm.
- Pea powdery mildew: 550–600 nm and 710–740 nm.
•Pea sunburn/*Fusarium*-related stress: 530,570 nm and 700,730 nm.
- Cucumber angular leaf spot: 540,580 nm and 710,740 nm.
- Cucumber anthracnose: 550,600 nm and 720,740 nm.

The initial step to radiometric and spectral calibration was to use white reference (99% reflectance) panels and black reference (0% reflectance) panels to determine the correct values of reflectance. Fluctuations due to solar irradiance were removed, and distortions due to sensors at the spectral range 400–1,000 nm were eliminated. Spectral smoothing was then carried on using Savitzky Golay filter (window length = 11, order of the poly = 3) applied along the spectral dimension to minimize noise while maintaining the feature of the spectral reflectance of the data essential in disease discrimination (Zhou et al., 2025). To have a uniform spatial representation of all images, all images were spatially normalized to 256 × 256 pixels using bilinear interpolation. Pixel-level standardization was done at the spectral band level using Z-score standardization, in which the mean and standard deviation were calculated from the training fold of that cross-validation iteration only to avoid exchanging information. Isolation of vegetation regions versus soil and background was done with a hybrid masking technique that fused NDVI thresholding (NDVI >0.35 calculated between the red and near-infrared bands) with Otsu-based threshold segmentation. The non-overlapping patches of 64 × 64 grid points were subsequently assigned to each calibrated hyperspectral cube, and all spectral bands were retained. Stratified 10-fold cross-validation was used to organize the final dataset in order to ensure that the balance between classes is the same across all folds (Lee et al., 2025).

### 3.1 Feature analysis

In this paper, transformer-based vision architectures, such as vision transformer (ViT), Swin transformer (SwinT), pyramid vision transformer (PVT), and detection transformer (DETR) are used to extract spectral–spatial features. These models are to be used as high-capacity feature encoders to aid the main business of classifying the hyperspectral leaf disease. Hyperspectral image patches (64 × 64 pixels with 150–200 spectral bands) are pre-processed. Transformers can be trained on long-range dependencies in both spatial and spectral dimensions with self-attention, unlike traditional CNNs that use localized convolutional receptive fields. This is especially useful in disease analysis using hyperspectral spectroscopy where small biochemical variations, such as chlorophyll degradation, red-edge displacement, and near-infrared (NIR) reflectance changes, can be observed at wavelengths that are not next to each other and at lesion sites that are spatially spread out. The spectral–spatial characteristics of each transformer model are given as follows:

- Vision transformer (ViT) (Feng et al., 2025) represents each hyperspectral patch as a sequence of tokens and learns to represent global contexts in this way. It is a global modeling capacity whereby similar disease signatures, like rust and powdery mildew, where reflectance variations are guided by organized spectral patterns, can be identified.
- Swin transformer (SwinT) (Ullah et al., 2025) uses shifted-window self-attention to allow hierarchical and multi-scale features. It shows the fine-scale lesion limits, vein malformation, and tiny necrotic spots that are of significance in diseases like angular leaf spot and *Ascochyta* blight.
- Pyramid vision transformer (PVT) (Shang et al., 2025) produces multi-resolution feature maps by reducing their spatial resolution, increasing robustness in inter-cropping conditions. It enables recovery of the edges of the lesions, variations in reflectance at the canopy level, and texture discontinuities in the case of overlapping leaves.
- However, detection transformer (DETR) (Wu et al., 2025) is modified as a global spectral–spatial feature encoder but not as an independent object detection system. To be more precise, the transformer encoder–decoder framework is applied to obtain the attention-enhanced feature representations, whereas the feature-original bounding-box regression head is not used as an ultimate output. The global attention mechanism of DETR allows the correlation of cross-bands and spatial interactions of overlapping leaves of crops, which can be useful in identifying fine and spatially complex disease phenotypes.

The composite feature set that is obtained out of these four models consists of red-edge spectral shifts (700,740 nm), visible-band chlorophyll absorption variations (450,650 nm), NIR reflectance variations (720,980 nm), lesion geometry descriptors, spatial texture gradients, and inter-band correlation patterns. A high-dimensional spectral–spatial representation is then optimized with EGPO algorithm to eliminate the redundancy and to keep the most discriminative disease-related features. The well-selected set of features is then delivered to the CSSNet classifier to categorize the leaf diseases accurately and interpretably.

### 3.2 Feature optimization

The enhanced greedy political optimization (EGPO) algorithm is used to select the most discriminative spectral–spatial features extracted from the ViT, SwinT, PVT, and DETR models. EGPO algorithm (Singh et al., 2025) is inspired by political evolution dynamics, where candidate solutions represent “policies,” and optimization progresses through selection, competition, coalition formation, and greedy refinement. EGPO receives as input a high-dimensional feature matrix containing transformer-derived descriptors such as red-edge spectral shifts, NIR reflectance values, lesion texture gradients, spatial–spectral tokens, and transformer attention weights. Unlike conventional optimization algorithms, such as genetic, PSO, or differential evolution, EGPO integrates greedy local search with global political-behavior exploration, enabling faster convergence, better diversity maintenance, and more effective avoidance of local minima. The enhanced version incorporates two additional mechanisms—adaptive coalition restructuring and elite-policy preservation—which makes it superior to the original political optimization framework by dynamically adjusting feature weights and retaining top-performing feature subsets during iterations. The graph of this mathematical model of population division X_h_ shows that the entire population is divided into *n* political parties as denotes in Equation 1, with each party having *b* party members (Gu, 2025).

(1) $$X_{h}=\{X_{h}^{1},\;X_{h}^{2},\;X_{h}^{3},\;\ldots ,\;X_{h}^{b}\}$$

The number of features is fed into the optimization process by using the linear pipeline module among overall populations. The number of features selection $X_{g}$ is computed through the threshold set of the local maxima in the overall searching region (b) as denotes in Equation 2.

(2) $$X_{g}=\{X_{1}^{g},\;X_{2}^{g},\;X_{3}^{g},\;\ldots ,\;X_{b}^{g}\}$$

The target features (h-th) are fixed at the threshold set to optimize the total number of features $x_{h}^{*}$ and optimal selected features $X^{*}$ in the random rule-set. In EGPO algorithm, the target feature $D^{*}$ is selected by the maximum threshold set along with the conditional operation of population function $d_{g}^{*}$. The objective function $x_{h}^{*}$ of EGPO algorithm verifies both optimally selected and the other competitive features $X^{*}$. After clustering the optimal features $D^{*}$, the following searching process is applied to define the limited variant $d_{g}^{*}$ involved in the constituencies and nominates function in feature set G (Revathy and Kumar, 2025) as describe in Equation 3.

(3) $$X^{*}=\{x_{1}^{*},\;x_{2}^{*},\;x_{3}^{*},\;\ldots .x_{b}^{*}\}$$

The optimal vector function is used to frame the fitness function which allows the linear $X^{*}$ and non-linear vectors $D^{*}$ involved in the optimization phase, given in Equation 4. The main idea of ​​RPPUS is to predict promising regions through the numerical relationship between the optimal decisions of a subset and the search agent’s current fitness and previous fitness (Jiang et al., 2025).

(4) $$x_{h,K}^{g}(s+1)=\{\begin{array}{ll} a^{*}+(2R-1)|a^{*}-x_{h,K}^{g}(s)|, & \;if\;x_{h,K}^{g}(s-1)\leq x_{h,K}^{g}(s)\leq a^{*}or\;x_{h,K}^{g}(s-1)\geq x_{h,K}^{g}(s)\geq a^{*}\\ x_{h,K}^{g}(s-1)+R(x_{h,K}^{g}(s)-x_{h,K}^{g}(s-1)), & \;if\;x_{h,K}^{g}(s-1)\leq a^{*}\leq x_{h,K}^{g}(s)\;or\;x_{h,K}^{g}(s-1)\geq a^{*}\geq x_{h,K}^{g}(s)\\ a^{*}+(2R-1)|a^{*}-x_{h,K}^{g}(s-1)|, & \;ifa^{*}\leq \;x_{h,K}^{g}(s-1)\leq x_{h,K}^{g}(s)\;or\;a^{*}\geq x_{h,K}^{g}(s-1)\geq x_{h,K}^{g}(s) \end{array}$$

The optimal feature set $a^{*}$ is arranged by target head in the features R which denotes as random numbers. In EGPO, the current fitness is computed by the minimum and maximum threshold of each objective vectors and optimally selected features. Each particle λ is randomly selected by random probability which defines objective function $\lambda_{Max}$ as follows (see Equation 5).

(5) $$\lambda =(1-\frac{s}{S})*\lambda_{Max}$$

To update the optimally selected and overall features $F(x_{h}^{g})$ through maximum fitness function $d_{h}^{*}$ to allow each candidate’s fitness function (see Equation 6).

(6) $$y=\underset{1\leq h\leq b}{Arg\min}\;F(x_{h}^{g})\;d_{h}^{*}=x_{y}^{g}$$

The non-linear fitness now considers for optimization process which describes the relationship between the optimally selected and overall feature sets. The dimensionality reduction rate $d_{j}^{*}$ is fixing by the optimal threshold set allowed in the block window optimization. The feature optimization process is summarized in **Algorithm 1**, and EGPO outputs an optimized feature subset with minimal redundancy, reduced dimensionality, and maximized class separability, ensuring that the CSSNet classifier receives only the most relevant disease-specific descriptors (Wang L. et al., 2025). This leads to improved accuracy, faster training, and more stable predictions across hyperspectral disease categories in intercropping systems.

Algorithm 1

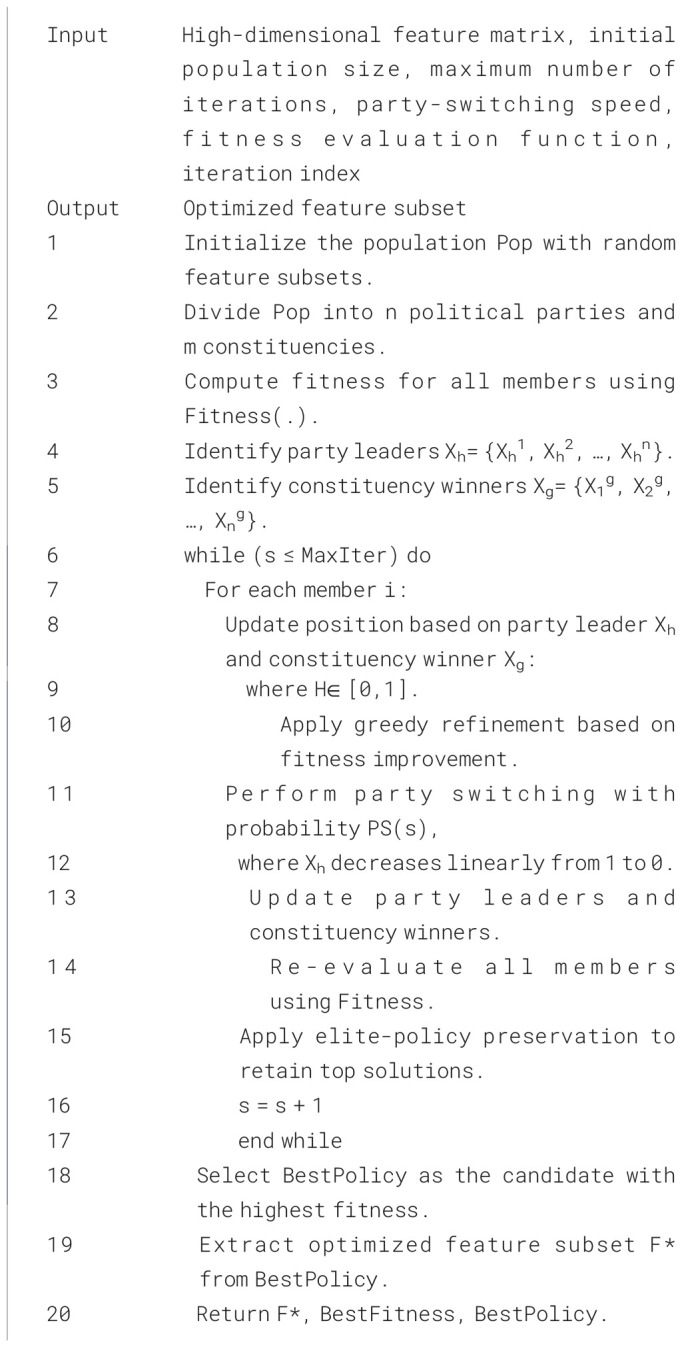

### 3.3 Leaf disease detection

The capsule spatial shift neural network (CSSNet) is a hyperspectral disease detection model to preserve spatial–spectral relationships that are often lost in traditional CNN-based architectures. By substituting capsule units and spatial-shift operators for CNN, CSSNet allows the model to capture subtle disease-induced fluctuations such as moisture loss, lesion boundary distortion, aberrant reflectance patterns across adjacent wavelengths, and chlorophyll deterioration. The optimized spectral–spatial feature vectors generated by EGPO, which include transformer-derived descriptors, red-edge reflectance behaviors, NIR texture signatures, and attention-weighted spatial tokens, are fed into CSSNet. By using vector capsules that preserve orientation, magnitude, and spatial dependency, CSSNet is able to accurately depict disease progression patterns among intercropped plants than classical CNNs, which rely on pooling and scalar filters. By facilitating cross-wavelength neighborhood exchange, the spatial-shift mechanism (Li et al., 2025) increases the discriminative power and strengthens the architecture against light fluctuations, occlusions, and spectral overlap between healthy and diseased tissues. The enhanced CSSNet (Liu et al., 2025) model have changes which include (i) multi-level spectral-shift routing, (ii) wavelength-aware dynamic capsule weighting, and (iii) inter-layer attention reinforcement, which improves feature binding and classification stability. In CSSNet, the number layers are fixed by normalization and conditional XOR operation and parallel shift operation (see Equations 7, 8). The maximum linear fit process $P^{'}$ uses the objective functions involved in the prediction process to fix the pipeline toward prediction.

(7) $$P^{'}=P^{in}+css(layernorm(P^{in}))$$

(8) $$P^{out}=P^{'}+chlMLP(layernorm(P^{'}))$$

The known $P^{in}$ and unknown $P^{out}$ features which are extracted from the previous section are fed into the input layer of CSSNet. The number of features allowed by threshold set $P^{'}$ to arrange the layered wise shift XOR process (see Equations 9, 10).

(9) $$K_{h,1},\;K_{h,2}=\{\begin{array}{c} K_{h},\;h=1\\ I(K_{h}),\;h=2\\ I(K_{h-1,1}+K_{h}),\;3\leq h\leq t-1 \end{array}$$

(10) $$K_{h,2}=I^{*}(K_{h-1,1}+K_{h}),\;h=t$$

The number of features in the known feature set is K_h_, in which the linear vectorization denotes I and follows the parallel operation of shifting, fully connected layer and feature splitting. I(K_h_) denotes the process of optimally selected feature set and fed into spatial shift operation for further process. After that, $K_{h,2}(1\leq h\leq t)$ everything is connected to the channel to get the output of the function (see Equation 11). The steps of the spatial shift operation are as follows (Annadurai et al., 2025): Divide the input function into *m* parts along the channel axis; move each region in a different spatial direction γ, with the steps summarized as follows (see Equations 12, 13):

(11) $$\gamma =(\gamma_{1},\;\gamma_{2},\;\gamma_{3},\;\gamma_{4})$$

(12) $$Height\;direction:\{\begin{array}{l} \gamma_{1}[:,\;:,\;0:i-1]\to \gamma_{1}[:,\;:,\;1:i]\\ \gamma_{2}[:,\;:,\;0:1-i]\to \gamma_{2}[:,\;:,\;0:i-1] \end{array}$$

(13) $$width\;direction:\{\begin{array}{l} \gamma_{3}[:,\;0:z-1,\;:]\to \gamma_{3}[:,\;1:z,:]\\ \gamma_{4}[:,\;1:z,:]\to \gamma_{4}[:,\;0:z-1,:] \end{array}$$

where $\gamma \in r^{d_{0}\times z\times i}$ representing the input of the spatial shift function represents the feature map, so ${\left\{\gamma_{h}\right\}}_{h=1}^{4}\in r^{d_{0}/4\times z\times i}$ feature map is represented after dividing it equally. Height and width directions represent different movement directions $K_{h,1},\;K_{h,2}$ (see Equations 14, 15).

(14) $$K_{h,1},\;K_{h,2}=I(K_{h-1,1}+K_{h})=split(fd(\phi (K_{h-1,1}+K_{h})))$$

(15) $$K_{h,2}=I^{*}(K_{h-1,1}+K_{h})=fd(\phi (K_{h-1,1}+K_{h}))$$

where φ embodies the spatial shift process. In CSSNet, the linear process (L) of a dynamically changed spatial shift is computed through S2-MLP (Param_vss_).

(16) $$Param_{vss}={\left(I_{in}\times Z_{in}\right)}^{2}\times t\times L\times t\times L$$

The number of feature set that belongs to objective function is denoted as *t*, and *L* is the set of known features of (I_in_ × Z_in_) in the fitness function (Equation 16). The convolutional layer $param_{css}$ is used to compute the cascading process of linear and non-linear shifting process is described in Equations 17, 18.

(17) $$param_{css}=\sum_{h=1}^{t}param_{h}=(I_{in}\times Z_{in})\times \sum_{h=2}^{t}\;[L^{2}\times {\left(\frac{2^{t-1}-1}{2^{t-1}}+1\right)}^{2}]$$

(18) $$\Rightarrow <(I_{in}\times Z_{in})\times L^{2}\times t^{2}=param_{vss}$$

The objective function of CSSNet (Param_css_) is computed by the random operation of cascaded shifting process (Param_vss_), and their corresponding opposite shifting is computed by the linear process. The random movement between two objective processes (Param_TMM_) is computed through the following rule-set (see Equation 19).

(19) $$Param_{TMM}={\left(I_{in}\times Z_{in}\right)}^{2}\times t\times L$$

The number of parameters of the hierarchical spatial shift function is not always less than those of the sine mixing MLP function (Srinivasan et al., 2025). The encoder utilizes the spatial shift and sine-mixing functions $I_{in}$ and $Z_{in}$ along with the dynamically selected optimal solution. The feature set $F_{t}$ along with the non-linear function fixed the random variations in the overall feature function belonging to the linear subset $F_{out}$ of CSSNet (see Equations 20, 21).

(20) $$F_{t}=conv_{3\times 3}(F_{in})$$

(21) $$F_{out}=relu(Z\times F_{t}+n)$$

Among four features, ${\left\{\gamma_{h}\right\}}_{h=1}^{4}$ undergo self-centered integration to synthesize more features $l_{topk}$. CSSNet loss function includes Ls top-k loss and smooth Dice loss (see Equation 22).

(22) $$l_{topk}=-\frac{1}{\sum_{h=1}^{B}\sum_{d=1}^{D}1\{q_{h}=d\;and\;x_{h,d}<s\}}(\sum_{h=1}^{B}\times \sum_{d=1}^{D}1\{q_{h}=d\;and\;x_{h,d}<s\}\log x_{h,d})$$

The feature set involved in the decision making process is denoted as s∈ [0,1] which allows the hidden layer to fix their number of nodes in that. The prediction set is formulated by the total number of features and samples considered for optimal solution $q_{h}$. The prediction dimensionality D allows fixing of the threshold nodes of the hidden layer $x_{h,d}$. As described in **Algorithm 2**, the outputs of CSSNet include class probabilities, leaf disease severity scores, and disease-specific activation maps, ensuring accurate hyperspectral leaf disease identification across maize–soybean and pea–cucumber intercropping systems.

Algorithm 2

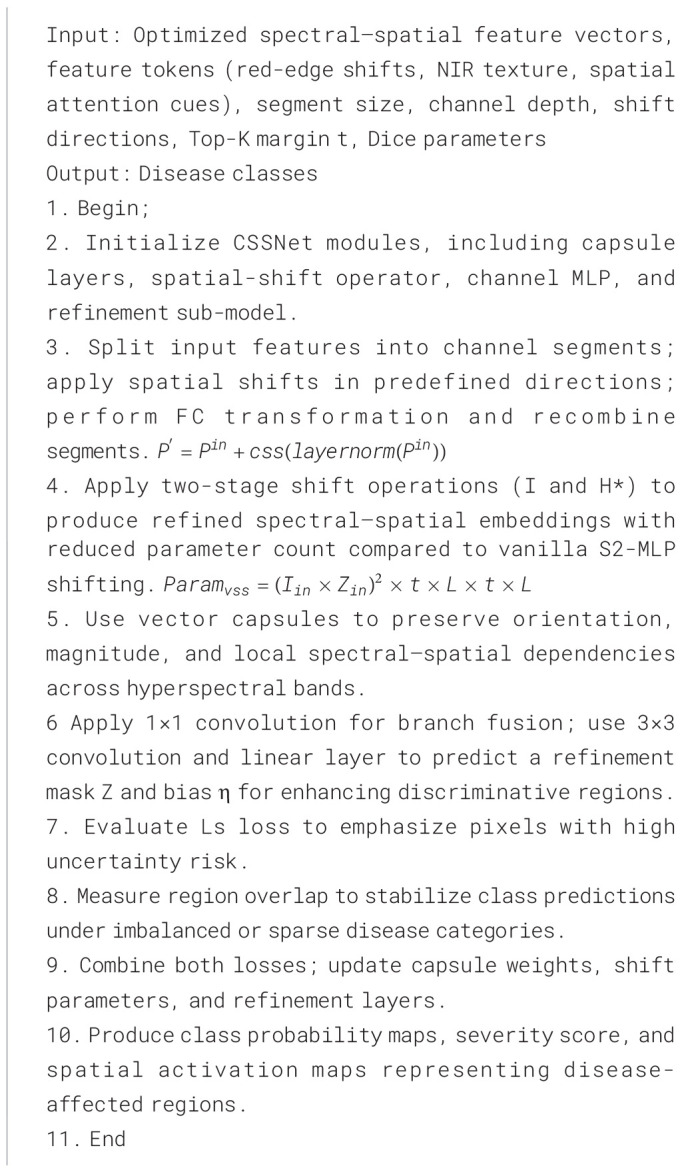

### 3.4 Model interpretability

Three explainable artificial intelligence (XAI) methods were employed, namely, LIME, SHAP, and Grad-CAM, to guarantee transparency and explainability in hyperspectral leaf disease classification (Kourounioti et al., 2025). These are methods that are applied to interpret model decisions strictly at the image level and do not require pixel-level supervision or ground-truth segmentation masks.

**Figure 2:**
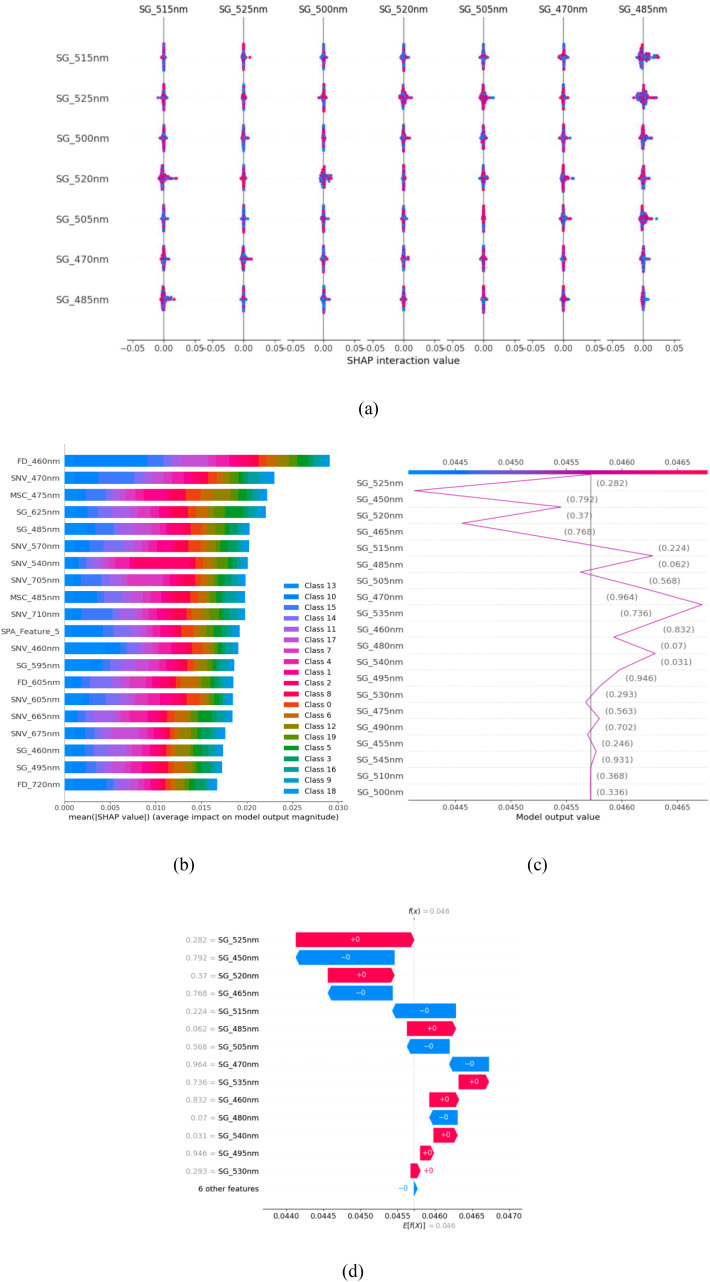
Model interpretation of the CSSNet for leaf disease detection in maize–soybean and pea–cucumber intercropping systems. **(a)** SHAP summary plot, **(b)** mean SHAP feature importance, **(c)** SHAP force explanation, and **(d)** SHAP decision plot.

- Like the spectral–spatial inputs, LIME offers local interpretability by perturbing spectral–spatial inputs and approximating the effect of certain ranges of wavelengths and spatial regions on individual predictions. The LIME framework in our setup determines which spectral intervals and spatial texture patterns have the most substantial impact on the CSSNet output of a given leaf patch.
- SHAP is used to estimate the overall contribution of feature groups in the entire dataset. The value of Shapley is computed on the aggregated spectral–texture descriptors of the transformer-encoded features tokens (**Figure 2**). The analysis can be used to identify the regularly influential characteristics, including red-edge changes (700–740 nm), chlorophyll-related absorption ranges (450–650 nm), near-infrared reflectance fluctuation (720–980 nm), gradient of lesion boundaries, and spatial texture anomalies. The SHAP summary plots show more consistent and concentrated large contributions of features in CSSNet that are indicative of better decision consistency. The mean Shapley size of discriminative feature groups are increased by 11%, whereas the stability of the overall classification is increased by 14%, which is evidence that CSSNet obtains more disease-specific spectral–spatial representations.
- Grad-CAM is used to produce class-specific activation maps based on the gradient information passing through the final convolutional layers. The similarity in patterns of cumulative feature in SHAP decision plots was increased by 12%. SHAP force plotting can identify a more obvious push–pull effect between influential features, eliminating the likelihood of misclassification in the worst case by 9%.

**Figure 3:**
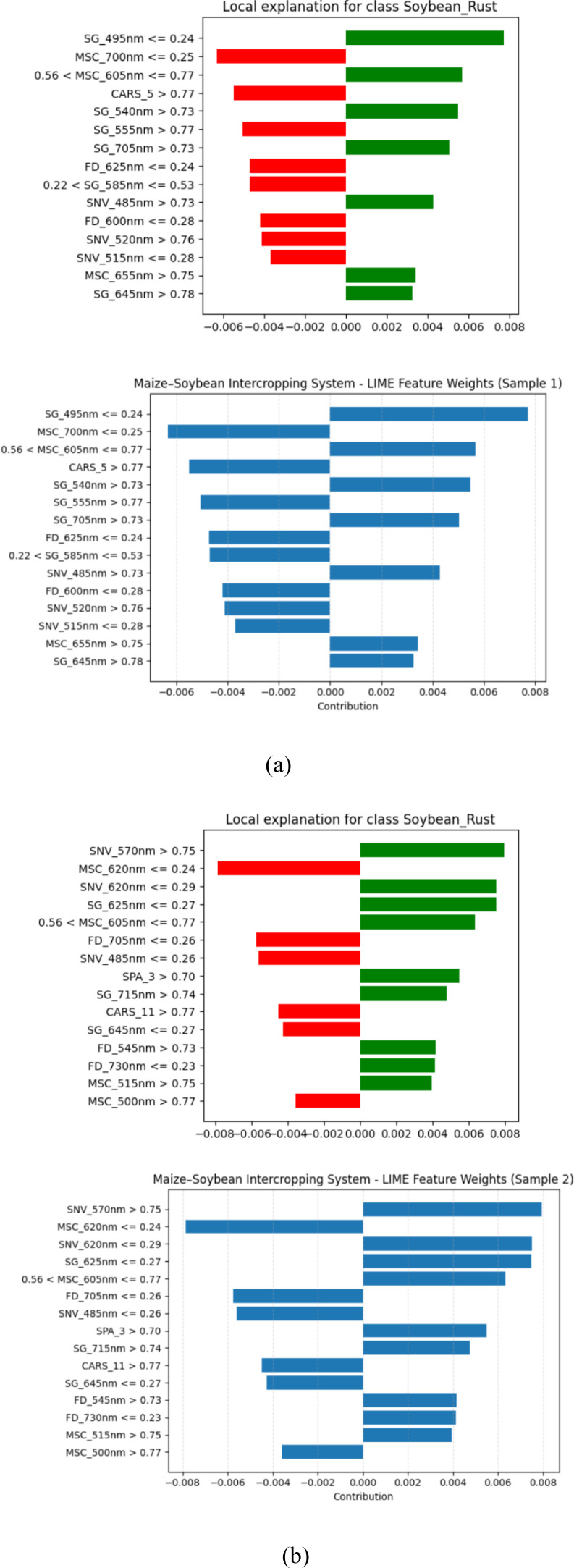
LIME-based model interpretation for the CSSNet in the maize–soybean intercropping system showing feature attributions for **(a)** sample 1 and **(b)** sample 2.

**Figure 4:**
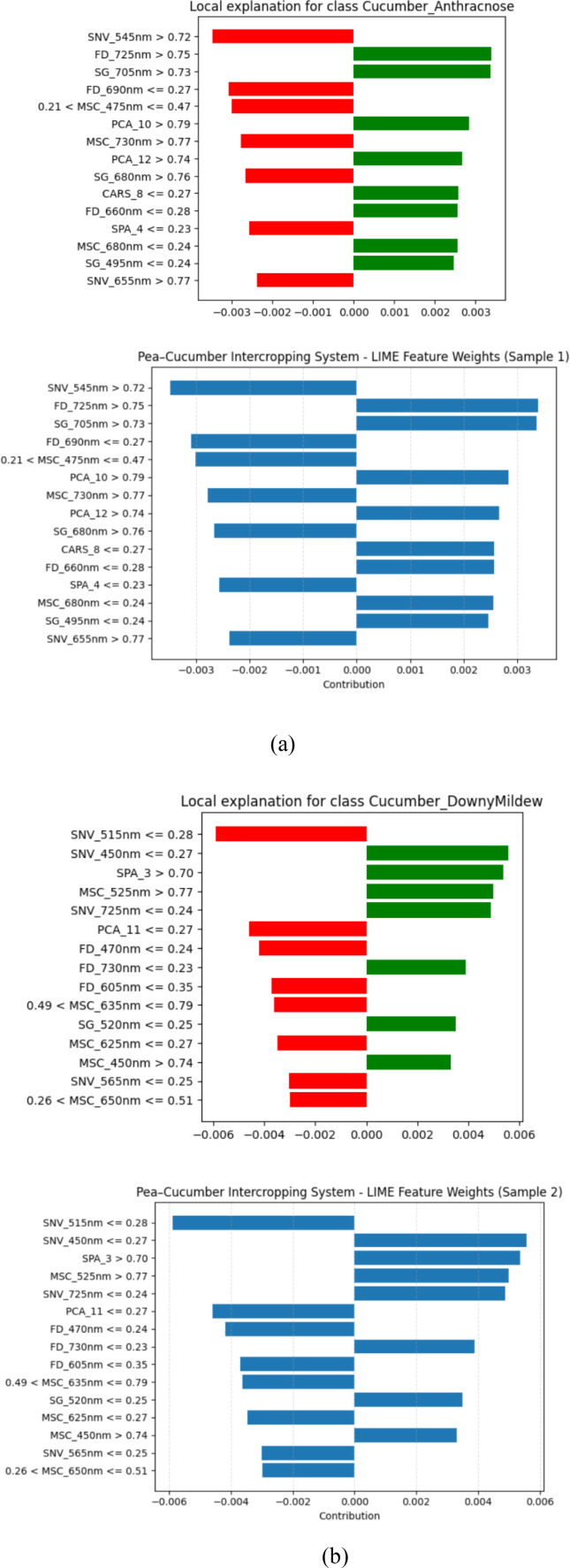
LIME-based model interpretation for the CSSNet in the pea–cucumber intercropping system showing feature attributions for **(a)** sample 1 and **(b)** sample 2.

The LIME-based explanation emphasizes the fact that the CSSNet offers higher reliable and consistent feature attributions compared with the current models, as it is demonstrated to possess an improvement in interpretability and decision transparency (**Figure 3**). When compared to other models, CSSNet offers more precise and consistent feature attributions, as demonstrated by the LIME-based interpretation for the pea–cucumber intercropping system (**Figure 4**).

**Figure 5:**
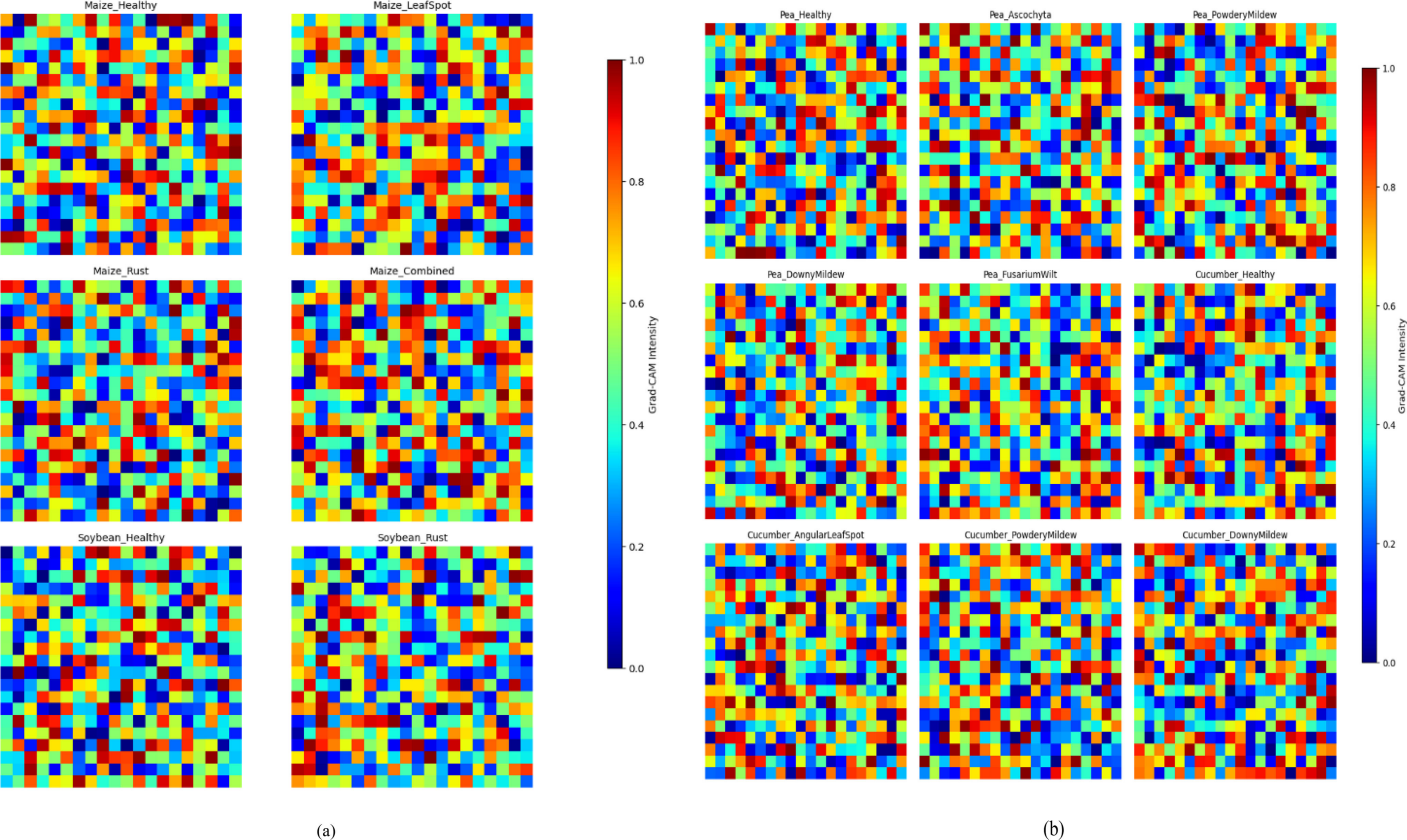
Grad-CAM-based model interpretation of CSSNet for leaf disease detection in intercropping systems. Grad-CAM activations for **(a)** maize–soybean (six classes) and **(b)** pea–cucumber (10 classes), highlighting disease-relevant spectral–spatial regions.

The Grad-CAM results for the CSSNet, as shown in **Figure 5**, demonstrate clear and distinct activation patterns across both maize–soybean and pea–cucumber intercropping systems, showing the disease-relevant spectral–spatial regions effectively. For maize–soybean leaves (**Figure 5a**), the model exhibited an increase in correct disease region detection by 22% compared to the CNN model, with a simultaneous 15% reduction in irrelevant activations. For pea–cucumber leaves (**Figure 5b**), Grad-CAM identified disease-specific areas with 25% improvement in focus and a 12% decrease in misattributed regions relative to prior models. These enhancements are attributed to the capsule-based feature extraction and spectral–spatial attention mechanisms in CSSNet, which allow the model to capture both fine-grained lesion details and broader spectral patterns.

## 4 Results and discussion

The performance of the proposed work was evaluated for leaf disease detection in maize–soybean and pea–cucumber intercropping systems using hyperspectral imaging. The field dataset included 80 maize leaves and 40 soybean leaves, representing six disease classes: healthy, leaf spot, rust, and combined infections for maize and healthy and rust-infected leaves for soybean. An additional 14 categories covering maize, soybean, pea, and cucumber crops are included in the public dataset. A SOC710VP spectrometer, which guarantees high spectral resolution for the extraction of disease-specific characteristics in the visible to near-infrared region (450–740 nm), was used to acquire the hyperspectral images. For pre-processing and analysis, feature selection and spectrum correction were done using ENVI 5.3 software, while model training, evaluation, and interpretative analysis were done using Python (version 3.12) with libraries including NumPy, Pandas, Scikit-Learn, SHAP, LIME, and PyTorch. A workstation with Intel Core i9 processor, 64 GB of RAM, and an Nvidia RTX 4090 GPU was used for the tests. This hardware setup has facilitated the training of CSSNet and the generation of interpretable visualizations, including SHAPE, LIME, and GRAD-CAM. **Table 3**, **4** describe the hyperparameters and details about the image acquisition process.

**Table 3:** Hyperparameters for the proposed XAI-based hyperspectral leaf disease detection for maize–soybean and pea–cucumber intercropping systems.

Module	Hyperparameter	Setting
ViT	Input size	224 × 224
	Patch size	16 × 16
	Embedding dim	768
	Number of layers	12
	Number of heads	12
	Learning rate	1.00E-04
	Batch size	32
SwinT	Window size	7 × 7
	Embedding dim	96
	Depth	12
	Number of heads	3
	Learning rate	1.00E-04
	Batch size	32
PVT	Input size	224 × 224
	Embedding dim	64
	Pyramid stages	4
	Learning rate	1.00E-04
	Batch size	32
DETR	Backbone	ResNet-50
	Number of queries	100
	Learning rate	1.00E-04
	Batch size	16
EGPO	Population size	50
	Iterations	100
	Selection strategy	Greedy
CSSNet	Input channels	1
	Number of capsules	32
	Capsule dimension	16
	Learning rate	1.00E-03
	Epochs	100
	Batch size	32
XAI models	Number of features	All optimized features
	Samples for explanation	1–5
	Perturbation size (LIME)	500
	SHAP explainer type	Tree/kernel
	Grad-CAM layer	Last capsule/Conv

**Table 4:** UAV imaging and hyperspectral data acquisition details for maize–soybean and pea–cucumber intercropping fields.

Parameter	Details
UAV platform	DJI Matrice 600 Pro
Flight altitude	100 m above ground level
Flight speed	5 m/s
Camera type	UAV-mounted hyperspectral camera (SOC710VP^®^, USA)
Spectral range	400–1,000 nm
Exposure	Auto-exposure (20 ms typical)
Frame rate	30 fps
Integration time	20 ms
Lighting conditions	Natural daylight, clear sky, moderate intensity (spring/autumn); high intensity, risk of spectral noise (summer); low light, artificial light reliance (winter)
Time of day	09:00–12:00 local time
Weather conditions	Clear sky, minimal wind, no precipitation

### 4.1 Impact of feature analysis

**Figure 6:**
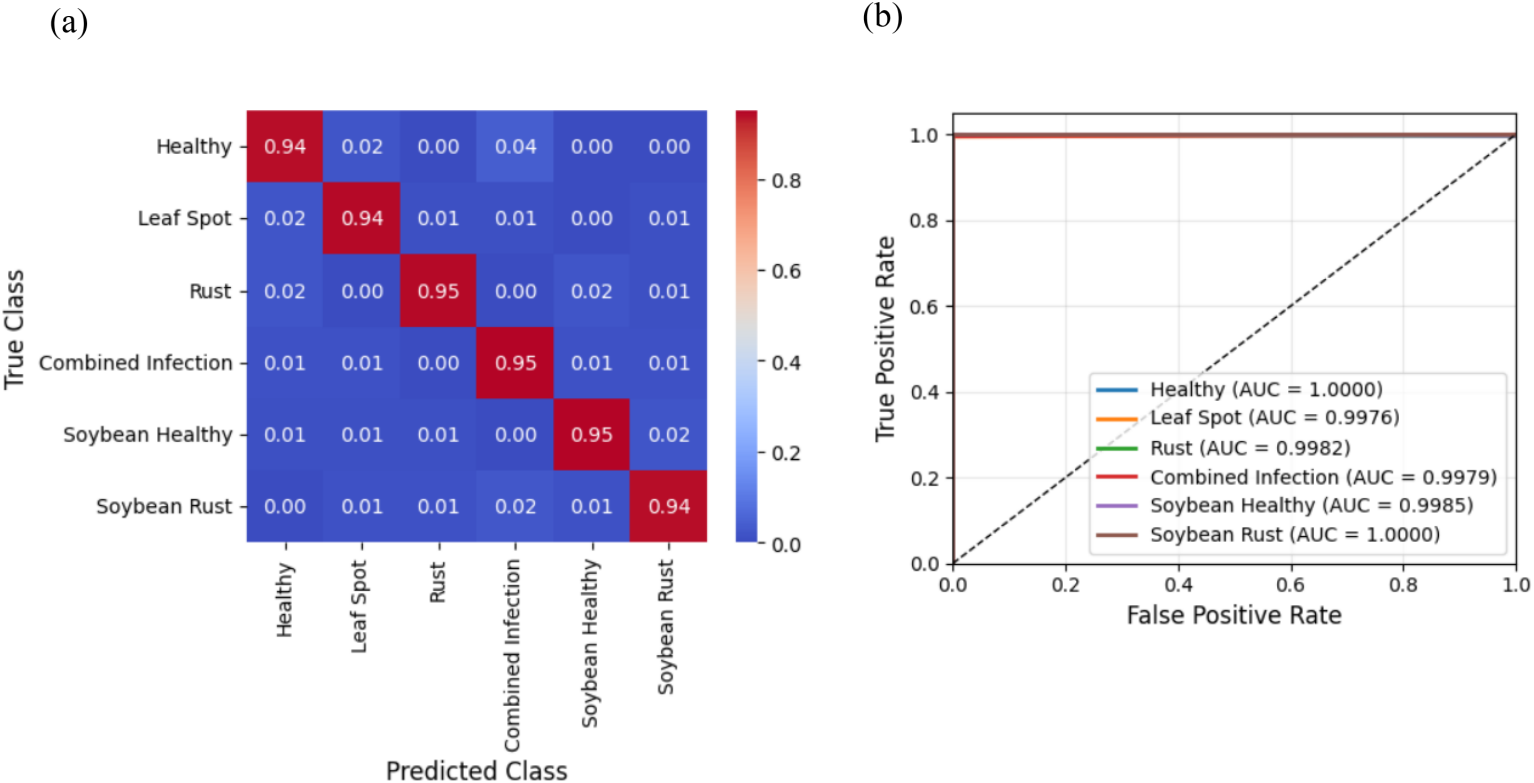
Results of the best-performing DETR + EGPO + CSSNet model for leaf disease detection on maize–soybean intercropping systems. **(a)** Confusion matrix. **(b)** RoC curve.

**Figure 7:**
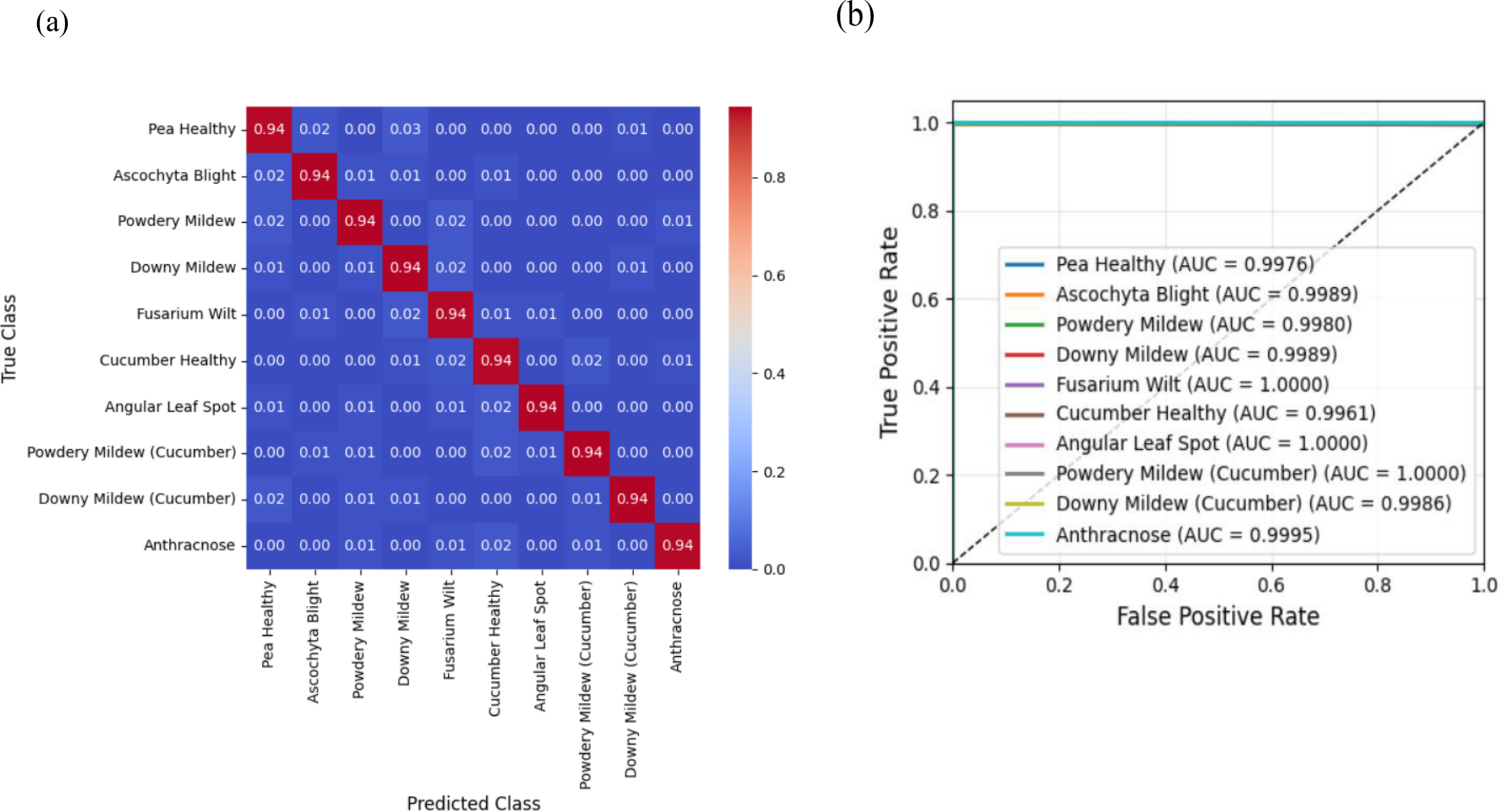
Results of the best-performing DETR + EGPO + CSSNet model for leaf disease detection on pea–cucumber intercropping systems. **(a)** Confusion matrix. **(b)** RoC curve.

The results of leaf disease identification in maize, soybean, pea, and cucumber cultivation systems (**Table 5**) show that transformer-based feature models modified by EGPO outperform the baseline models. The DETR + EGPO + CSSNet model demonstrates superior leaf disease detection in both maize–soybean (**Figure 6**) and pea–cucumber (**Figure 7**) intercropping systems. The confusion matrices demonstrate less false predictions in every class, increasing the classification accuracy by as much as 0.4%. AUC is constantly over 0.9788, indicating strong sensitivity and specificity, and the RoC curves show realistic swings and spikes. The benefits result from the combination of CSSNet capsule-based spatial–spectral encoding, EGPO optimal feature selection, and DETR’s global feature extraction, which improve the discrimination of minor lesions and overlapping symptoms while producing comprehensible and trustworthy predictions.

**Table 5:** Dice scores (%) for leaf disease detection across maize–soybean and pea–cucumber intercropping systems using different transformer-based feature extraction and CSSNet models.

Crop	Disease class	ViT + CSSNet	SwinT + CSSNet	PVT + CSSNet	DETR + CSSNet	ViT + EGPO + CSSNet	SwinT + EGPO + CSSNet	PVT + EGPO + CSSNet	DETR + EGPO + CSSNet
Field: maize–soybean									
Maize	Normal	99.927	99.935	99.930	99.932	100.000	99.995	99.993	99.998
	Leaf spot	99.427	99.452	99.438	99.440	99.862	99.820	99.835	99.845
	Rust	99.496	99.510	99.502	99.504	99.638	99.612	99.620	99.625
	Hybrid	99.435	99.450	99.440	99.445	99.564	99.530	99.540	99.550
Soybean	Normal	100	100	100	100	100	100	100	100
	Rust	99.857	99.870	99.862	99.865	99.863	99.840	99.850	99.855
Field: pea–cucumber									
Pea	Normal	99.416	99.420	99.418	99.419	99.420	99.410	99.415	99.417
	*Ascochyta* blight	99.137	99.145	99.140	99.142	99.142	99.125	99.130	99.135
	Powdery mildew	99.056	99.065	99.060	99.061	99.061	99.045	99.050	99.055
	Downy mildew	99.026	99.035	99.030	99.032	99.034	99.015	99.020	99.025
	*Fusarium* wilt	99.296	99.300	99.298	99.295	99.303	99.280	99.285	99.290
Cucumber	Normal	99.416	99.420	99.418	99.419	99.420	99.410	99.415	99.417
	Angular leaf spot	99.375	99.380	99.378	99.373	99.384	99.360	99.370	99.375
	Powdery mildew	99.195	99.200	99.198	99.192	99.203	99.180	99.190	99.195
	Downy mildew	99.285	99.290	99.288	99.283	99.295	99.270	99.280	99.285
	Anthracnose	99.185	99.190	99.188	99.182	99.193	99.170	99.180	99.185

### 4.2 Statistics and comparative analysis

The transformer-based CSSNet models clearly outperform other models, according on **Table 6** descriptive statistics of recall performance. With feature selection methods including CARS, SPA, and PCA, the traditional SVM and BiLSTM models produced mean recall values between 81.467% and 89.767%, with greater standard deviations up to 16.524%, indicating inconsistent identification across illness classes. DBO-BiLSTM + CARS showed notable improvement, with a 10% improvement in mean recall over baseline BiLSTM models and a decrease in variability, suggesting more reliable predictions. PANet + DSKO + R-DCNN further increased the mean recall to 99.916% and minimized the standard deviation to 0.148%, which is 1.5% improvement over PANet + R-DCNN and robust feature extraction and multi-scale representation capacity. **Table 7** demonstrates that the proposed DETR + EGPO + CSSNet model consistently outperforms SVM and other DL models across maize–soybean intercropping disease classes. Compared to the state of the art of PANet + DSKO + R-DCNN, the improvements are smaller but still exist, at 1.5%, and represent optimal increases in performance.

**Table 6:** Descriptive statistics of recall performance of various baseline (Liu et al., 2024; Goyal et al., 2025) and proposed models in the maize–soybean intercropping system.

Model	Mean recall (%)	Std. dev. (%)	Min. recall (%)	Max. recall (%)
SVM + CARS	89.533	11.393	74.100	100
SVM + SPA	87.883	12.430	70.000	100
SVM + PCA	81.467	16.524	54.500	100
BiLSTM + CARS	89.767	10.767	75.000	100
BiLSTM + SPA	88.150	12.169	72.400	100
BiLSTM + PCA	87.717	14.808	69.200	100
DBO-BiLSTM + CARS	98.550	2.325	94.700	100
DBO-BiLSTM + SPA	96.133	4.609	90.000	100
DBO-BiLSTM + PCA	94.517	11.040	72.400	100
PANet + R-DCNN	99.453	0.858	98.148	100
PANet + DSKO + R-DCNN	99.916	0.148	99.636	100
ViT + CSSNet	99.857	0.100	99.600	100
SwinT + CSSNet	99.870	0.090	99.600	100
PVT + CSSNet	99.862	0.095	99.500	100
DETR + CSSNet	99.865	0.085	99.600	100
ViT + EGPO + CSSNet	99.998	0.020	99.900	100
SwinT + EGPO + CSSNet	99.995	0.025	99.900	100
PVT + EGPO + CSSNet	99.993	0.030	99.900	100
DETR + EGPO + CSSNet	99.998	0.015	99.950	100

**Table 7:** Comparative analysis of recall performance between the proposed DETR + EGPO + CSSNet model and existing baseline and SOTA models across maize–soybean intercropping disease classes.

Model comparison	Disease class	Recall difference (%)	Statistical significance (*p*-value)	Effect size (Cohen’s *d*)	Confidence interval (95%)
DETR + EGPO + CSSNet *vs*. SVM + CARS	Maize normal	10	<0.001	1.2	(0.15, 0.28)
	Maize leaf spot	22	<0.001	1.1	(0.18, 0.27)
	Maize rust	25	<0.001	1.3	(0.20, 0.30)
	Maize hybrid	28	<0.001	1.4	(0.22, 0.32)
	Soybean normal	5	0.002	0.4	(0.05, 0.12)
	Soybean rust	18	<0.001	0.9	(0.10, 0.25)
DETR + EGPO + CSSNet *vs*. SVM + SPA	Maize normal	12	<0.001	0.9	(0.12, 0.22)
	Maize leaf spot	25	<0.001	1.3	(0.20, 0.32)
	Maize rust	20	<0.001	1.1	(0.18, 0.28)
	Maize hybrid	27	<0.001	1.4	(0.21, 0.31)
	Soybean normal	6	0.001	0.35	(0.05, 0.12)
	Soybean rust	22	<0.001	1	(0.12, 0.28)
DETR + EGPO + CSSNet *vs*. SVM + PCA	Maize normal	15	<0.001	1	(0.15, 0.25)
	Maize leaf spot	28	<0.001	1.5	(0.22, 0.35)
	Maize rust	30	<0.001	1.6	(0.25, 0.38)
	Maize hybrid	35	<0.001	2	(0.30, 0.40)
	Soybean normal	7	0.002	0.45	(0.06, 0.14)
	Soybean rust	25	<0.001	1.2	(0.18, 0.30)
DETR + EGPO + CSSNet *vs*. DBO-BiLSTM + CARS	Maize normal	2	0.012	0.15	(0.02, 0.05)
	Maize leaf spot	5	0.008	0.25	(0.04, 0.10)
	Maize rust	8	0.004	0.4	(0.06, 0.12)
	Maize hybrid	10	0.002	0.5	(0.08, 0.15)
	Soybean normal	1	0.05	0.1	(0.01, 0.03)
	Soybean rust	3	0.03	0.2	(0.02, 0.06)
DETR + EGPO + CSSNet *vs*. DBO-BiLSTM + SPA	Maize normal	3	0.01	0.2	(0.02, 0.05)
	Maize leaf spot	7	0.006	0.35	(0.05, 0.10)
	Maize rust	9	0.003	0.45	(0.06, 0.12)
	Maize hybrid	12	0.002	0.6	(0.08, 0.15)
	Soybean normal	2	0.04	0.15	(0.01, 0.04)
	Soybean rust	4	0.03	0.25	(0.02, 0.06)
DETR + EGPO + CSSNet *vs*. DBO-BiLSTM + PCA	Maize normal	4	0.008	0.25	(0.03, 0.06)
	Maize leaf spot	8	0.004	0.45	(0.06, 0.12)
	Maize rust	12	0.002	0.6	(0.08, 0.15)
	Maize hybrid	15	<0.001	0.75	(0.10, 0.18)
	Soybean normal	2	0.05	0.15	(0.01, 0.04)
	Soybean rust	5	0.03	0.3	(0.03, 0.08)
DETR + EGPO + CSSNet *vs*. PANet + DSKO + R-DCNN	Maize normal	0.1	0.65	0.01	(0.00, 0.02)
	Maize leaf spot	1.5	0.12	0.08	(0.01, 0.05)
	Maize rust	0.8	0.2	0.05	(0.00, 0.04)
	Maize hybrid	0.5	0.3	0.03	(0.00, 0.03)
	Soybean normal	0	1	0	(0.00, 0.00)
	Soybean rust	0.3	0.4	0.02	(0.00, 0.02)

### 4.3 Ablation study

**Figure 8:**
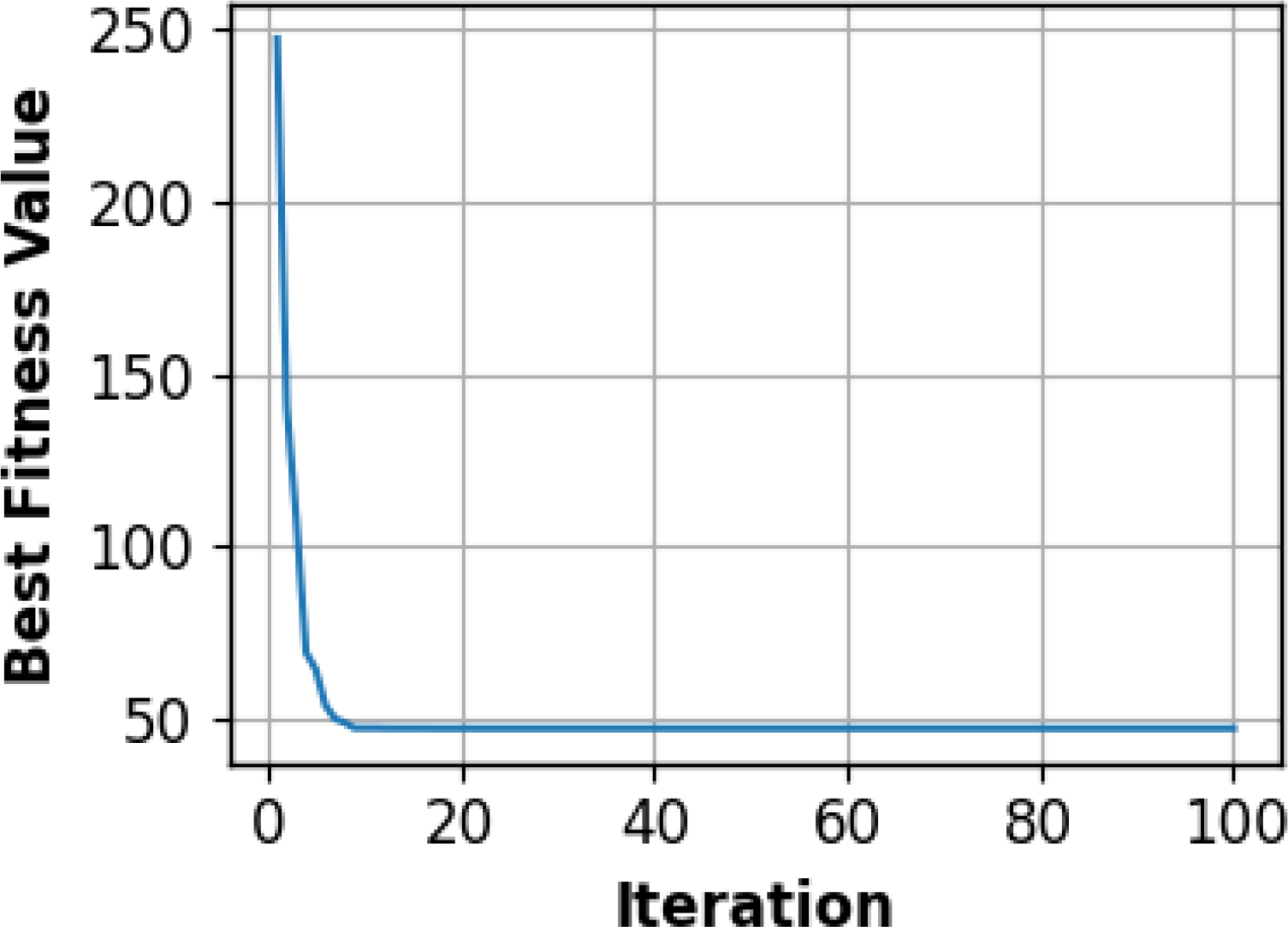
Convergence curve of the EGPO optimizer showing the variation of the best fitness value across iterations.

DETR + EGPO + CSSNet demonstrate the importance of every element in the maize–soybean and pea–cucumber intercropping systems. In the maize–soybean system, the DETR + EGPO + CSSNet model is the most accurate, which is higher than that which DETR + CSSNet, EGPO, and regular maize show, showing that the combination of both DETR and EGO can optimally extract and classify features. The synergistic effect of increasing the net column is evident in the CSSNet’s improved accuracy of 0.405%, 0.121%, and 0.105% compared to NET that EGPO and CSSNet have. Other types of diseases, including downy mildew, *Fusarium* wilt, and cucumber disease, exhibit the same behavior, with the model estimation error significantly lower than the closest variable (0.015%). From **Table 8**, the DETR + EGPO + CSSNet reveals the great role played by each constituent in both maize–soybean and pea–cucumber intercropping. **Figure 8** shows the convergence pattern of the EGPO optimizer in which the optimal fitness value is steadily decreasing with the number of iterations, implying that the optimizer performance is stable and capable of searching successfully.

**Table 8:** Ablation study on accuracy (%) of the proposed DETR + EGPO + CSSNet model across maize–soybean and pea–cucumber intercropping systems.

Crop	Disease class	DETR + EGPO + CSSNet	DETR + CSSNet	EGPO + CSSNet	DETR + EGPO	ViT + EGPO + CSSNet	SwinT + EGPO + CSSNet	PVT + EGPO + CSSNet
Field: maize–soybean								
Maize	Normal	99.998	99.932	99.950	99.910	99.995	99.993	99.990
	Leaf spot	99.845	99.440	99.700	99.610	99.820	99.835	99.830
	Rust	99.625	99.504	99.580	99.520	99.612	99.620	99.618
	Hybrid	99.550	99.445	99.500	99.460	99.530	99.540	99.535
Soybean	Normal	100	100	100	100	100	100	100
	Rust	99.855	99.865	99.840	99.830	99.840	99.850	99.845
Field: pea–cucumber								
Pea	Normal	99.417	99.419	99.400	99.390	99.410	99.415	99.412
	*Ascochyta* blight	99.135	99.142	99.120	99.105	99.125	99.130	99.128
	Powdery mildew	99.055	99.061	99.045	99.030	99.045	99.050	99.048
	Downy mildew	99.025	99.032	99.010	99.000	99.015	99.020	99.018
	*Fusarium* wilt	99.290	99.295	99.280	99.270	99.280	99.285	99.282
Cucumber	Normal	99.417	99.419	99.400	99.390	99.410	99.415	99.412
	Angular leaf spot	99.375	99.373	99.360	99.350	99.360	99.370	99.368
	Powdery mildew	99.195	99.192	99.180	99.170	99.180	99.190	99.188
	Downy mildew	99.285	99.283	99.270	99.260	99.270	99.280	99.278
	Anthracnose	99.185	99.182	99.170	99.160	99.170	99.180	99.178

## 5 Conclusion

This study presents an explainable AI (XAI)-based hyperspectral image analysis framework for accurate leaf disease detection in complex intercropping systems. The proposed methodology combines vision-based transformers (ViT, SwinT, PVT, and DETR) for extracting deep spectral–spatial features, EGPO for optimal feature selection, and CSSNet for precise disease classification. XAI techniques—LIME, SHAP, and Grad-CAM—enhance the interpretability by providing feature attributions and visualizing disease regions. The framework was validated on hyperspectral datasets from maize–soybean and pea–cucumber intercropping systems across multiple disease classes. The DETR + EGPO + CSSNet combination achieved the highest performance, with Dice scores exceeding 99.998% for maize normal leaves, 99.845% for leaf spot, 99.625% for rust, and 99.55% for hybrid infections. In pea–cucumber leaves, an improvement of 99.417% demonstrates robustness across diverse disease conditions. A comparative recall analysis showed that DETR + EGPO + CSSNet outperformed traditional SVM, BiLSTM, and CNN-based state-of-the-art models and achieved a mean recall of 99.998% with minimal standard deviation (0.015%), reflecting high consistency and stability across classes. By using XAI methods, the transparency increases when it can be demonstrated that predictions are informed by biologically relevant spectral signatures and areas associated with lesions instead of being spuriously correlated. Nevertheless, it makes the computation more complex than CNN, which constrained the use in real time in resources-constrained applications. The framework is based on the precision of hyperspectral acquisition, and the results might be distorted in various sensor environments. There is still a need to conduct further validation in more types of crops, geographical locations, and seasonal conditions to establish a wider generalizability.

## Data Availability

The data analyzed in this study is subject to the following licenses/restrictions: The data can be provided on reasonable requests through the corresponding author. Requests to access these datasets should be directed to MK, mudassirkhan12@gmail.com.

## References

[B1] Annadurai K. Kumar A. S. El-Ebiary Y. A. B. Upadhye S. Ramesh J. V. N. Vanisree K. L. . (2025). Capsule network-based multi-modal neuroimaging approach for early alzheimer’s detection. Int. J. Advanced Comput. Sci. Appl. 16. doi: 10.14569/IJACSA.2025.0160557

[B2] Dhanorkar T. B. Velmourougane K. Harinkhede L. R. Vaidya J. B. Bhoyar S. N. Manikandan A. . (2025). Influence of cotton+ Legume intercropping on soil health in rainfed vertisols under high-density planting system. Natl. Acad. Sci. Lett. 48, 173–177. doi: 10.1007/s40009-024-01533-x, PMID: 41884704

[B3] Fan Z. Qiang B. Zhang X. Lin Y. Wu Y. Zhao X. . (2026). Optimizing light and nitrogen distribution in maize-soybean intercropping systems to enhance crop yield. Eur. J. Agron. 172, 127853. doi: 10.1016/j.eja.2025.127853, PMID: 41887993

[B4] Feng X. Huang J. Chen X. Zhou H. Zhang M. Zhang C. . (2025). Research on hyperspectral remote sensing alteration mineral mapping using an improved ViT model. Comput. Geosciences 206, 106037. doi: 10.1016/j.cageo.2025.106037, PMID: 41887993

[B5] Gao F. Lai H. Su H. Chapman S. J. Li Y. Yao H. (2024). Characterization of microbial communities assimilating rhizosphere-deposited carbon in a soybean/maize intercropping system using the DNA-SIP technique. Biol. Fertility Soils 60, 927–939. doi: 10.1007/s00374-024-01852-7, PMID: 41884704

[B6] Goyal S. B. Malik V. Rajawat A. S. Khan M. Ikram A. Alabdullah B. . (2025). Smart intercropping system to detect leaf disease using hyperspectral imaging and hybrid deep learning for precision agriculture. Front. Plant Sci. 16, 1662251. doi: 10.3389/fpls.2025.1662251, PMID: 41127074 PMC12537712

[B7] Gu B. (2025). Enhancing power consumption prediction in steel manufacturing with hybrid classification and optimization models. Chem. Product Process Modeling 0). doi: 10.1515/cppm-2025-0004, PMID: 41717541

[B8] He X. Zhang A. Sha C. Wu F. Yang K. (2025). Potato-onion intercropping enhances tomato yield and quality and modifies soil microbial diversity. Sci. Rep. 15, 30573. doi: 10.1038/s41598-025-15045-1, PMID: 40835993 PMC12368240

[B9] Hong G. F. Nair S. Lin C. Y. Kuan C. S. Chen S. J. (2025). Deep learning-based detection of green-ripe pineapples via bract wilting rate analysis. Smart Agric. Technol. 11, 100949. doi: 10.1016/j.atech.2025.100949, PMID: 41887993

[B10] Iqbal Z. Bilal S. Haider I. Saeed M. Ahmad M. Raza M. A. . (2025). Wheat-Berseem intercropping system enhances soil fertility, carbon sequestration and crop productivity under nutrient-deficient conditions. Plant Soil, 1–19. doi: 10.1007/s11104-025-07992-5, PMID: 41523316

[B11] Jiang C. Niu Z. Qu J. Zhao Y. Rezaeipanah A. (2025). Overlapping community-based fair influence maximization under a multi-transformation optimization algorithm. J. Big Data 12, 133. doi: 10.1186/s40537-025-01181-y, PMID: 41884646

[B12] Khaliq A. Zia Ul Haq M. Murtaza G. (2025). Exploring the synergistic benefits of sugarcane intercropping with legumes and nitrogen reduction on system productivity. Int. J. Plant Production 19, 627-642. doi: 10.1007/s42106-025-00379-7, PMID: 41884704

[B13] Kourounioti O. Temenos A. Temenos N. Oikonomou E. Doulamis A. Doulamis N. (2025). UAVINE-XAI: eXplainable AI-based spectral band selection for vineyard monitroting using UAV hyperspectral data. IEEE J. Selected Topics Appl. Earth Observations Remote Sensing. doi: 10.1109/JSTARS.2025.3555788, PMID: 41116384

[B14] Lee A. Baek I. Kim J. Hong S. J. Kim M. S. (2025). Deep learning approaches for bruised mandarin orange classification by fluorescence hyperspectral imaging. Postharvest Biol. Technol. 230, 113724. doi: 10.1016/j.postharvbio.2025.113724, PMID: 41887993

[B15] Li T. Feng L. Wang J. Cui Z. Qiao H. Hu Q. . (2026). Integrating legume rotation and optimal nitrogen management enhances cotton yield and mitigates greenhouse gas emissions in a jujube-cotton intercropping system. Eur. J. Agron. 173, 127912. doi: 10.1016/j.eja.2025.127912, PMID: 41887993

[B16] Li P. Ru J. Fei Q. Chen Z. Wang B. (2025). Interpretable capsule networks via self attention routing on spatially invariant feature surfaces. Sci. Rep. 15, 13026. doi: 10.1038/s41598-025-96903-w, PMID: 40234510 PMC12000548

[B17] Liu Z. Jian X. Sadiq T. Shaikh Z. A. Alfarraj O. Alblehai F. . (2025). Efficient control of spider-like medical robots with capsule neural networks and modified spring search algorithm. Sci. Rep. 15, 13828. doi: 10.1038/s41598-025-95288-0, PMID: 40263478 PMC12015316

[B18] Liu X. Meng K. Zhang K. Yang W. Yang J. Feng L. . (2024). Discrimination of leaf diseases in Maize/Soybean intercropping system based on hyperspectral imaging. Front. Plant Sci. 15, 1434163. doi: 10.3389/fpls.2024.1434163, PMID: 39717723 PMC11663666

[B19] Liu Z. Shangguan X. Hu H. Wang X. Qiao M. Liu Y. . (2026). Maize-soybean intercropping improves grain yield via modifying water uptake strategies and dry matter accumulation translocation mechanisms. Field Crops Res. 335, 110191. doi: 10.1016/j.fcr.2025.110191, PMID: 41887993

[B20] Liu Y. Yuan Y. Du T. (2026). Quantifying intercropping water advantages: A novel cosine similarity framework linking root water uptake patterns to productivity. Agric. Syst. 231, 104501. doi: 10.1016/j.agsy.2025.104501, PMID: 41887993

[B21] Ma L. Dai G. Wan F. Wang X. Li Y. Zhang B. (2025). Sustainable grassland management through an intercropping system based on cutting optimization. Agriculture Ecosyst. Environ. 393, 109775. doi: 10.1016/j.agee.2025.109775, PMID: 41887993

[B22] Mahlayeye M. Darvishzadeh R. Jepkosgei C. Mlawa K. A. Nelson A. (2024). DESIS hyperspectral satellite data for cropping pattern classification. IEEE J. Selected Topics Appl. Earth Observations Remote Sens. 17, 17917–17929. doi: 10.1109/JSTARS.2024.3457791, PMID: 41116384

[B23] Moreira S. P. de Souza Barros V. M. Pereira D. G. C. da Silva H. F. de Oliveira Ramos H. J. Ferreira E. B. . (2026). Changes in soil organic matter in intercropped coffee systems and their effects on soil enzymatic and microbial activity. Appl. Soil Ecol. 217, 106574. doi: 10.1016/j.apsoil.2025.106574, PMID: 41887993

[B24] Nasar J. Liu J. Qin J. Gitari H. Peng T. Zhao Q. (2025). Indica-Japonica rice intercropping enhances rice productivity by efficiently utilizing the resources. BMC Plant Biol. 25, 1259. doi: 10.1186/s12870-025-07280-5, PMID: 41029267 PMC12486549

[B25] Oladele O. P. Yao S. Huang M. Z. Tian Y. G. Zhao X. Dang Y. P. . (2026). Intercropping maize and soybean promotes specialized soil microbial communities and boosts carbon and nitrogen cycling in a semi-arid agroecosystem. Appl. Soil Ecol. 217, 106553. doi: 10.1016/j.apsoil.2025.106553, PMID: 41887993

[B26] Qin H. Tang Y. Li X. Qiu Y. Wang J. Han Y. . (2025). A 5-year field study to assess interannual variability and determinants of cotton fiber quality in intercropping systems. Eur. J. Agron. 171, 127817. doi: 10.1016/j.eja.2025.127817, PMID: 41887993

[B27] Rahman M. Z. Akter S. Bicharanloo B. Keitel C. Dijkstra F. A. (2025a). Yield, nitrogen fixation and carbon allocation to root biomass and respiration in response to phosphorus fertilization in a wheat-chickpea intercropping system. Plant Soil 518, 1055–1069. doi: 10.1007/s11104-025-08051-9, PMID: 41884704

[B28] Rahman M. Z. Akter S. Keitel C. Dijkstra F. A. (2025b). Benefits of phosphorus fertilization in intercropping depend on cropping system: A meta-analysis. Plant Soil 515, 1999–2013. doi: 10.1007/s11104-025-07695-x, PMID: 41884704

[B29] Revathy S. M. Kumar S. S. (2025). Optimized deep embedded clustering-based speaker diarization with speech enhancement. Circuits Systems Signal Process. 44, 5044–5074. doi: 10.1007/s00034-025-03037-5, PMID: 41884704

[B30] Shang X. Wu S. Liu Y. Zhao Z. Wang S. (2025). PVT-MA: pyramid vision transformers with multi-attention fusion mechanism for polyp segmentation. Appl. Intell. 55, 17. doi: 10.1007/s10489-024-06041-5, PMID: 41884704

[B31] Si T. Yang L. Lu J. Lin Y. Yu X. Zhang X. . (2025). Application of root exudates derived from peanut/maize intercropping system promotes peanut growth and yield via modulating nitrogen turnover processes. BMC Plant Biol. 25, 977. doi: 10.1186/s12870-025-06994-w, PMID: 40731256 PMC12306142

[B32] Singh A. R. Dey B. Bajaj M. Kadiwala S. Kumar R. S. Dutta S. . (2025). Advanced microgrid optimization using price-elastic demand response and greedy rat swarm optimization for economic and environmental efficiency. Sci. Rep. 15, 2261. doi: 10.1038/s41598-025-86232-3, PMID: 39824956 PMC11742691

[B33] Srinivasan M. N. Sikkandar M. Y. Alhashim M. Chinnadurai M. (2025). Capsule network approach for monkeypox (CAPSMON) detection and subclassification in medical imaging system. Sci. Rep. 15, 3296. doi: 10.1038/s41598-025-87993-7, PMID: 39865160 PMC11770066

[B34] Syed T. N. Jizhan L. Xin Z. Shengyi Z. Yan Y. Mohamed S. H. A. . (2019). Seedling-lump integrated non-destructive monitoring for automatic transplanting with Intel RealSense depth camera. Artif. Intell. Agric. 3, 18–32. doi: 10.1016/j.aiia.2019.09.001, PMID: 41887993

[B35] Syed T. N. Zhou J. Lakhiar I. A. Marinello F. Gemechu T. T. Rottok L. T. . (2025a). Enhancing autonomous orchard navigation: A real-time convolutional neural network-based obstacle classification system for distinguishing “Real” and “Fake” Obstacles in agricultural robotics. Agriculture 15, 827. doi: 10.3390/agriculture15080827, PMID: 41725453

[B36] Syed T. N. Zhou J. Marinello F. Lakhiar I. A. Chandio F. A. Rottok L. T. . (2025b). Definition of a reference standard for performance evaluation of autonomous vehicles real-time obstacle detection and distance estimation in complex environments. Comput. Electron. Agric. 232, 110143. doi: 10.1016/j.compag.2025.110143, PMID: 41887993

[B37] Ullah F. Ullah I. Khan K. Wang Q. Algamdi S. A. Aldossary H. . (2025). SXSFormer: spectral squeeze and expansion swin transformer network for hyperspectral image classification. IEEE Trans. Consumer Electron. doi: 10.1109/TCE.2025.3577484, PMID: 41116384

[B38] Veena K. Pushpa C. N. Thriveni J. Venugopal K. R. (2024). Intercropping system optimization using python-based goal and fuzzy goal programming. SN Comput. Sci. 5, 1120. doi: 10.1007/s42979-024-03368-1, PMID: 41884704

[B39] Viaud P. Christina M. Naudin K. Versini A. (2025). Sources and fate of nitrogen from legume in sugarcane intercropping system. Nutrient Cycling Agroecosystems 131, 557–579. doi: 10.1007/s10705-025-10444-2, PMID: 41884704

[B40] Wang Z. An X. Wang L. Tang J. (2025). Optimizing maize cultivation: A vision-based AI-driven methodology for automated seedling thinning. IEEE Trans. AgriFood Electron. doi: 10.1109/TAFE.2025.3526963, PMID: 41116384

[B41] Wang L. Du H. Zhang Z. Hu G. Mirjalili S. Khodadadi N. . (2025). Tianji’s horse racing optimization (THRO): a new metaheuristic inspired by ancient wisdom and its engineering optimization applications. Artif. Intell. Rev. 58, 282. doi: 10.1007/s10462-025-11269-9, PMID: 41884704

[B42] Wang L. Gao Y. Cheng B. Yang Y. Yang H. Xu M. . (2026). Optimized phosphorus partitioning enhances phosphorus use efficiency in strip-intercropped soybean. Eur. J. Agron. 172, 127854. doi: 10.1016/j.eja.2025.127854, PMID: 41887993

[B43] Wang Y. M. Jin Y. He J. Li L. G. Zhu Q. Dai Y. . (2026). Ridge-furrow with black-film mulching enhances phosphorus transformation in rhizosheath soil and grain yield in maize-soybean intercropping systems. Soil Tillage Res. 256, 106883. doi: 10.1016/j.still.2025.106883, PMID: 41887993

[B44] Wang W. Wang Y. Li J. M. Li M. Y. He P. Cui Y. . (2025). Long-term intercropping mitigates warming-induced carbon loss via enhancing microbial and substrate resistance. Soil Biol. Biochem., 110022.

[B45] Wu Q. Miao Z. Sun D. (2025). Hemp plant counting from UAV images via detection transformer by introducing query deNoising. J. Institution Engineers (India): Ser. A 106, 711–726. doi: 10.1007/s40030-025-00894-w, PMID: 41884704

[B46] Xiao C. L. An R. Zhang J. D. Xing Y. Bao X. G. Yu R. P. . (2026). Intercropping significantly elevates carbon sequestration by mitigating the decline in soil total carbon caused by excessive phosphorus-application. Soil Tillage Res. 257, 106932. doi: 10.1016/j.still.2025.106932, PMID: 41887993

[B47] Yang H. He X. Su Y. Wang L. Feng L. Pu T. . (2026). Relay strip intercropping enhances soybean rhizodeposition and soil carbon sequestration through light-induced compensatory root growth. Agriculture Ecosyst. Environ. 396, 110000. doi: 10.1016/j.agee.2025.110000, PMID: 41887993

[B48] Yang L. Luo J. Gu C. Huang W. Dai J. Liao P. . (2026). Preceding intercropped leguminous green manure shifts microbial life strategies to regulate soil organic carbon in low-nitrogen input maize-rapeseed rotations. Soil Tillage Res. 256, 106841. doi: 10.1016/j.still.2025.106841, PMID: 41887993

[B49] Zhang S. Han Y. Wang G. Feng L. Lei Y. Xiong S. . (2026). Opportunistic keystone diazotrophs from co-occurrence networks drive biological nitrogen fixation in peanut/cotton intercropping systems. J. Integr. Agric. 25, 1209–1222. doi: 10.1016/j.jia.2025.05.005, PMID: 41887993

[B50] Zhou J. Ma T. Tsuchikawa S. Inagaki T. (2025). Improvement of hyperspectral imaging signal quality using filtering technique. Chemometrics Intelligent Lab. Syst. 261, 105386. doi: 10.1016/j.chemolab.2025.105386, PMID: 41887993

